# Adaptation potential of the copepod *Eurytemora affinis* to a future warmer Baltic Sea

**DOI:** 10.1002/ece3.6267

**Published:** 2020-05-15

**Authors:** Konrad Karlsson, Monika Winder

**Affiliations:** ^1^ Department of Arctic Biology University Centre in Svalbard Svalbard Norway; ^2^ Department of Ecology, Environment and Plant Sciences Stockholm University Stockholm Sweden

**Keywords:** genotype by environment interaction, global warming, life history, multiple stressors, phenotypic plasticity, trade‐off

## Abstract

To predict effects of global change on zooplankton populations, it is important to understand how present species adapt to temperature and how they respond to stressors interacting with temperature. Here, we ask if the calanoid copepod *Eurytemora affinis* from the Baltic Sea can adapt to future climate warming. Populations were sampled at sites with different temperatures. Full sibling families were reared in the laboratory and used in two common garden experiments (a) populations crossed over three temperature treatments 12, 17, and 22.5°C and (b) populations crossed over temperature in interaction with salinity and algae of different food quality. Genetic correlations of the full siblings’ development time were not different from zero between 12°C and the two higher temperatures 17 and 22.5°C, but positively correlated between 17 and 22.5°C. Hence, a population at 12°C is unlikely to adapt to warmer temperature, while a population at ≥17°C can adapt to an even higher temperature, that is, 22.5°C. In agreement with the genetic correlations, the population from the warmest site of origin had comparably shorter development time at high temperature than the populations from colder sites, that is, a cogradient variation. The population with the shortest development time at 22.5°C had in comparison lower survival on low quality food, illustrating a cost of short development time. Our results suggest that populations from warmer environments can at present indirectly adapt to a future warmer Baltic Sea, whereas populations from colder areas show reduced adaptation potential to high temperatures, simply because they experience an environment that is too cold.

## Graphical abstract

Here, we ask whether the Baltic Sea copepod *Eurytemora affinis* can adapt to future climate warming. Our results on genetic correlations show that *E. affinis* can adapt to a future high temperature, 22.5°C, via indirect selection at 17°C; however, in a cold environment, 12°C, populations are unlikely to adapt to warmer temperatures of 17 and 22.5°C. These results suggest that populations from warmer environments can at present adapt to a future warmer Baltic Sea, whereas populations from colder areas have reduced adaptation potential to high temperatures, simply because they experience an environment that is too cold.

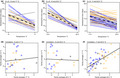

## 1 INTRODUCTION

Global warming affects the distribution and ecology of populations (De Meester, Stoks, & Brans, 2018; Kratina, Greig, Thompson, Carvalho‐Pereira, & Shurin, 2012; Parmesan, 1996; Urban et al., 2016); however, the magnitude of its effect will ultimately depend on the populations’ adaptation potential to changing environmental conditions (Aitken, Yeaman, Holliday, Wang, & Curtis‐McLane, 2008; Merilä & Hendry, 2014; Urban, Richardson, & Freidenfelds, 2014). Both genetic and plastic variation may facilitate retention of populations that otherwise would go extinct or be in need of migration to colder areas (Davis & Shaw, 2001; Foden et al., 2013; Hughes et al., 2003). Evidence exists for some populations that have adapted to high temperatures (Lonsdale & Levinton, 1985; Yampolsky, Schaer, & Ebert, 2014), and if species exhibit adaptations at present, it is likely that they will in the future as well (Merilä & Hendry, 2014; Stoks, Geerts, & De Meester, 2014). However, there are still important topics to address on how species may adapt to climate change, such as how contemporary populations can adapt to future conditions. This can be estimated by quantifying indirect selection between present and future environments, which is revealed by the sign and strength of genetic correlations. Moreover, many studies focus on one factor at a time (Todgham & Stillman, 2013), and hence, much less is known about the effect of multiple factors interacting simultaneously with temperature (Stoks et al., 2014).

Adaptive potential is essentially genetic variance (Foden et al., 2013; Urban et al., 2014), from which a series of estimates related to selection and adaptation can be calculated. For example, if a trait is measured on groups of full siblings, the proportion of phenotypic variance that is caused by between‐group variance is the heritability, which is a predictor between *direct* selection and adaptation. More so, if the same trait is measured across different environments, the correlation of between‐group variances in each environment is the genetic correlation, which is a predictor between *indirect* selection and adaptation. Indirect selection depends on the sign of the correlation and may either reinforce, antagonize, or have no effect on adaptation (Etterson & Shaw, 2001). For example, if a high value of the same trait is of benefit in two environments, such as extant and future conditions, and the genetic correlation between the trait in both environments is positive, then selection at extant conditions will render a high value also in future conditions through indirect selection. Hence, genetic correlations are highly relevant for inferences of local adaptation and for the adaptation potential of populations.

The difference in trait value between two or more environments is the phenotypic plasticity; this is an environmentally induced change in the phenotype that enables a single genotype to respond differently to various environmental conditions (Via et al., 1995). Plasticity may also vary between genotypes in response to the environment, that is, an interaction between the genotype and the environment (Falconer & Mackay, 1996; Lee, 2002; Saltz et al., 2018). The variance between genotypes in different environments may reveal if selection in one environment will have a correlated, indirect, response in another environment. Hence, there is a formal link between the genotype by environment interaction and the genetic correlation (Falconer, 1990; Falconer & Mackay, 1996).

For zooplankton, development time is a useful trait for studying adaptation since it is intimately connected to fitness, with a shorter development time increasing the exponential fitness parameter *r* and hence population growth (Allan, 1976; Lewontin, 1965). Species in seasonal environments that produce several generations over the year, should in theory, benefit if the development time is as short as possible when conditions are favorable (Allan, 1976; Kingsolver & Huey, 2008; Roff, 1980). Body size and fecundity are also important for population growth rates of zooplankton; however, they are relatively less important than the time lag between generations (Allan, 1976).

Typically, populations with a short development time are comparably smaller when they reach maturity than populations with longer development time (Kingsolver, Massie, Ragland, & Smith, 2007; Merilä, Laurila, & Lindgren, 2004; Sniegula, Golab, Drobniak, & Johansson, 2016). Hence, a fitness trade‐off between size (via fecundity) and development may influence the evolution of thermal reaction norms. Although, exceptions from the typical trade‐off exists where populations can maintain both fast development and large size at maturity (Gotthard, Nylin, & Wiklund, 1994; Tang, He, Chen, Fu, & Xue, 2017). However, maximizing both traits involves increased growth rates and can result in higher susceptibility to starvation (Gotthard et al., 1994; Stoks, Block, & McPeek, 2006). Thus, overcoming one trade‐off includes a new trade‐off. This is important in a scenario where other stressors may change in addition to temperature and indirectly affect organisms’ response to temperature.

The calanoid copepod *Eurytemora affinis* is at places one of the dominating zooplankton species in terms of number and mass in both freshwater and coastal estuaries, and hence an important grazer and prey for plankton feeding fish (Diekmann, Clemmesen, John, Paulsen, & Peck, 2012; Hernroth & Ackefors, 1979; Rajasilta, Hänninen, & Vuorinen, 2014). In the Baltic Sea, *E. affinis* forms large transitory populations that typically peak in late summer (Hernroth & Ackefors, 1979). Given this opportunistic (*r*) life strategy, it is expected that *E. affinis* has a development time that is as short as physiologically possible when conditions are favorable. *Eurytemora affinis* consists of a species complex with a widespread distribution in the northern Hemisphere (Lee, 2016). Within the complex, both development time and body size differ between populations (Karlsson, Puiac, & Winder, 2018; Karlsson & Winder, 2018). More so, the populations are highly variable in diverse traits, such as morphology, habitat use, ecological effects, and salinity tolerance (Favier & Winkler, 2014; Karlsson & Winder, 2018; Lee, Remfert, & Gelembiuk, 2003). Clades and lineages are also spread outside their native range because of maritime traffic and introduced into other environments (Sukhikh, Souissi, Souissi, & Alekseev, 2013; Winkler, Souissi, Poux, & Castric, 2011). However, the rapid adaptations recorded in this species complex support that even invasive populations might be locally adapted to their new environments (Lee, 2002; Lee, Posavi, & Charmantier, 2012; Lee, Remfert, & Chang, 2007).

The Baltic Sea is one of the marine areas with the highest recorded temperature increase during the past century (Meier, 2015), and climate change may increase precipitation in the catchment area possibly leading to lower salinity and changes in food web structure (Lefebure et al., 2013; Meier, 2015). The Baltic Sea spans over a large latitudinal and ecological gradient and consists of different basins that vary in temperature, salinity, and food web structure (i.e., trophic states, terrestrial organic matter) (Andersen et al., 2017; Larsson, Elmgren, & Wulff, 1985; Lefebure et al., 2013; Lehmann, Getzlaff, & Harlaß, 2011). The copepod *E. affinis* is widely distributed in the Baltic Sea, and populations are thus subjected to different environmental conditions and to different selection pressures depending on their geographical position.

The aim in this study was to investigate if the copepod *E. affinis* may adapt to a future warmer Baltic Sea. For this, a quantitative genetics approach was used, with related individuals (full siblings) crossed over different temperatures in common garden experiments. *Eurytemora affinis* was further exposed to different temperatures in combination with different salinity and food type to explore interactions of multiple stressors. For this, populations that originate from areas of different temperature, salinity, and primary production were compared to investigate local adaptations and trade‐offs.

## 2 MATERIALS AND METHODS

### 2.1 Study populations and rearing conditions

**Figure 1:**
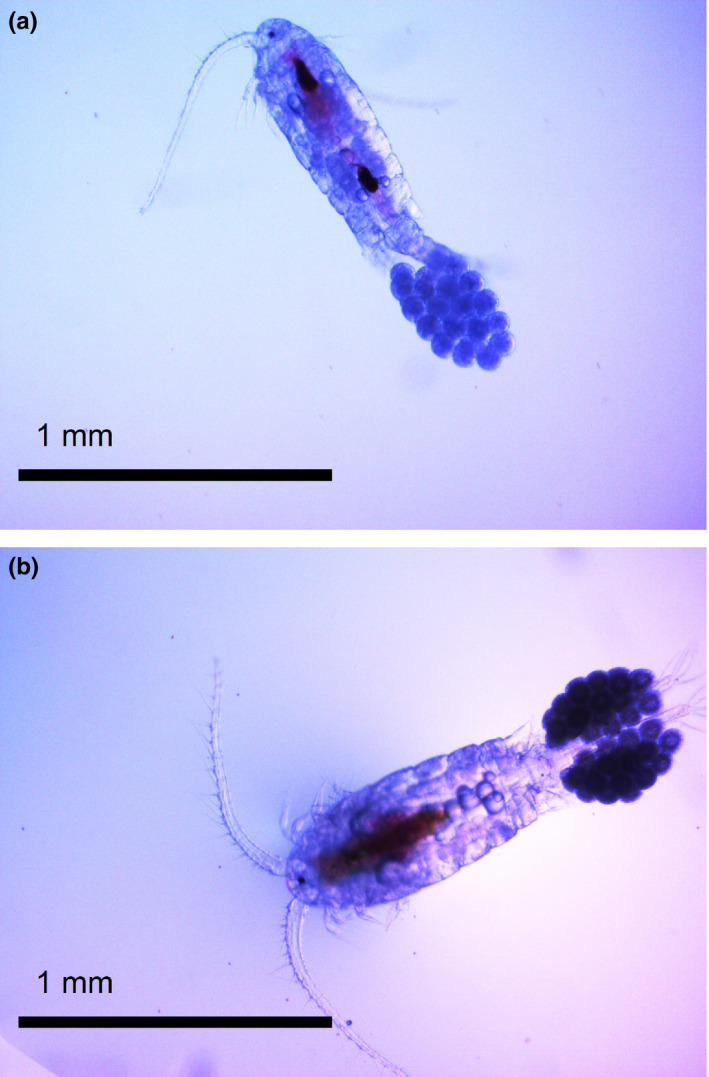
*Eurytemora affinis* females from the STHLM (a) and GOR (b) sampling stations with a prosome length of 768 and 869 µm, respectively; this is the average length based on 50 individual measurements per population; in addition, the populations differ in shape where the GOR population is wider (Karlsson & Winder, 2018)

*Eurytemora affinis* were collected with 90 µm vertical tow nets in autumn 2014 from the Bothnian Bay (BB, monitoring station F3A5, 65°10.14’, 23°14.41’), the Gulf of Riga–Pärnu Bay (GOR, 58°21.67’, 24°30.83’), and the Stockholm Archipelago–Askö (STHLM, monitoring station B1, 58°48.19’, 17°37.52’). The GOR population has in previous studies shown to develop to adult faster and at a larger size (Figure 1) than the STHLM population (Karlsson et al., 2018; Karlsson & Winder, 2018). Copepods were transported to the department in cooled conditions and placed in a cold room where temperature gradually increased up to 17°C over the course of several days.

In the laboratory, a minimum of 300 individuals were sorted out from each location and put into cultures maintained at 17°C and salinity of seven practical salinity units (PSU, g/kg). Tap water was used for the stock cultures and breeding, and the water was circulated in an aquarium for approximately 1 week with gravel from a small stream, making it more habitable for aquatic organisms. The water was then mixed with Instant Ocean^TM^ to reach appropriate salinity. The copepod cultures were fed two types of Cryptophytes: *Rhodomonas salina* and *Rhinomonas nottbecki*. The copepods were reared at a relatively high temperature, 17°C, at which *E. affinis* reproduces relatively rapidly and could undergo many generations at common conditions. Before the experiments, all populations had gone through at least three generations, likely many more, in common conditions in order to control for environmental and maternal effects (Sanford & Kelly, 2011). The choice of salinity was based on the survival of the food source, *R. salina*, which did not grow well at lower salinities.

### 2.2 Analyses of environmental conditions

Environmental data on temperature, salinity, and chlorophyll‐*a* were obtained from the Swedish Meteorological and Hydrological Institute (SMHI) for the BB (station F3) and STHLM (station B1) sites and the International Council for the Exploration of the Sea (ICES) for the GOR site. The GOR population was not sampled at a monitoring station, and hence, data are from the geographical cut‐off: highest lat, lon 58°35.00’, 24°47.17’; lowest 58°02.50’, 24°17.17’. All available observations from depth ≤10 m between the years 1993 and 2018 were used for the analyses. The data were analyzed as nine separate generalized additive models (GAM), one for each population and parameter combination, and a smooth function was applied to the linear predictor day of year. The GAM models were fitted by the use of package mgcv (Wood, 2004, 2011). The predicted fitted values and 95% CI were used to assess any “significant” differences in parameters between sites. Furthermore, projections of sea surface temperature increase in the Baltic Sea for the years 2069–2098, relative to a baseline of 1978–2007, are available from Meier (2015). The projected values were added to predicted temperature values estimated from a baseline time period of 1993–2007, as high‐resolution monitoring data were unavailable at the sampling sites before 1993, and this was done in order to plot future site‐specific temperatures. In addition, monitoring data on *Eurytemora* sp. abundance from SMHI and ICES were analyzed with GAMs in order to visualize timing of population abundance peaks over the same time period as for the environmental data. Here, abundances of different life stages from each sample were added up and predicted over day of year.

### 2.3 Common garden experiments

Two common garden experiments were designed; the first experiment took place in April–June 2015 and the second in January–March 2016. The first experiment included three populations BB, GOR, and STHLM with in total 37 families and 273 individuals that matured to adults (Table 1). Here, three temperature treatments 12, 17, and 22.5°C were used, and these were in the range of what *E. affinis* populations experience in the Baltic Sea during the summer period. For the first experiment, the salinity was at 7 PSU and *R. salina* was used as food.

**Table 1:** The number of full sibling families and individuals in each treatment combination of temperature, salinity, and food condition. The number of families and individuals is given for the life stage nauplii (N1, newly hatched) and for those who survived to adults (C6, last and final stage)

Population	Temperature (°C)	Salinity	Food	Experiment	Families, N1	Individuals, N1	Families, C6	Individuals, C6
BB	12	7	*Rhodomonas salina*	One	14	54	8	17
BB	17	7	*R. salina*	One	12	53	7	15
BB	22.5	7	*R. salina*	One	13	54	8	11
GOR	12	7	*R. salina*	One	11	58	10	28
GOR	17	7	*R. salina*	One	11	57	11	47
GOR	22.5	7	*R. salina*	One	11	57	11	48
STHLM	12	7	*R. salina*	One	12	57	11	37
STHLM	17	7	*R. salina*	One	12	60	11	31
STHLM	22.5	7	*R. salina*	One	12	53	12	39
GOR	17	2	*Cryptomonas* sp.	Two	13	47	13	27
GOR	17	2	*Rhinomonas nottbecki*	Two	13	39	11	25
GOR	17	7	*R. nottbecki*	Two	13	46	11	30
GOR	22.5	2	*Cryptomonas* sp.	Two	13	46	9	19
GOR	22.5	2	*R. nottbecki*	Two	13	44	13	36
GOR	22.5	7	*R. nottbecki*	Two	13	39	12	25
STHLM	17	2	*Cryptomonas* sp.	Two	14	33	14	23
STHLM	17	2	*R. nottbecki*	Two	15	32	15	24
STHLM	17	7	*R. nottbecki*	Two	15	29	12	15
STHLM	22.5	2	*Cryptomonas* sp.	Two	15	34	12	20
STHLM	22.5	2	*R. nottbecki*	Two	14	27	10	21
STHLM	22.5	7	*R. nottbecki*	Two	15	30	12	18

For the second experiment, two populations GOR and STHLM were used, and in total 28 families and 283 individuals that matured to adults (Table 1). Here, two temperatures 17 and 22.5°C, two salinities 2 and 7 PSU, and two types of food *Cryptomonas* sp. and *R. nottbecki* were used. Both food type and salinity were crossed over temperature and population; however, food and salinity were not fully factorial because *Cryptomonas* sp. could be cultured in 2 but not 7 PSU. In contrast, *R. nottbecki* was cultured in salinity 2 and 7 PSU and was therefore used as food in both salinity treatments. In comparison with *R. salina* from the first experiment, *R. nottbecki* is in size (*c.* 12 µm long and 5 µm wide), shape, growth rate, and color very similar (personal observation) and we assumed they are of similar and high food quality. *Cryptomonas* sp. is slightly bigger than the other two species (*c*. 20 µm long and 10 µm wide). All three species are members of the phylum Cryptophyta, *R. salina* and *R. nottbecki* belong to the family Pyrenomonadaceae, while *Cryptomonas* sp. belongs to Cryptomonadaceae. Cryptophytes are in general known as a good food sources for calanoid copepods leading to a short development time and high egg production (Knuckey, Semmens, Mayer, & Rimmer, 2005; Koski, Breteler, & Schogt, 1998).

To obtain full sibling clutches, *E. affinis* mature males and copepodite females (that would later undergo sexual maturity) were paired up in 15 ml cylinders at 17°C, and this procedure ensured that only one male fertilized the eggs as copepod females may store sperm (Allan, 1976). Once the egg sacks became visible, the eggs were separated with an injection needle under a stereomicroscope and placed into 10 ml vials, with 1–3 eggs per vial depending on clutch size. Eggs from each full sibling clutch (family) were split across temperature (experiment one), as well as temperature*food (experiment two) and temperature*salinity (experiment two) with two vials for each family and treatment combination. Thereby, family lines were put in specific environments, which make it possible to separate between genetic and environmental variance.

For the experiments, the aquarium water was filtered through a 0.7 µm pore size filter (WhatmanTM GF/F) before adding food and copepod eggs to the vials. The algae were observed every day to ensure that they remained in a healthy state during the experiment, which is reflected in the color of the water and is pink‐red for *R. salina* and *R. nottbecki* and green for *Cryptomonas *sp. In some vials, the algae culture died and was replaced as soon as it was detected. The algae suspension in the experimental vials had a concentration of approximately 200,000 cells per ml, and this concentration is well above ad libitum for *E. affinis* (Ban, 1994). The vials were put in racks in temperature incubators (INKU‐line from WVR) with a precision of ±0.5°C.

Development time from nauplii (newly hatched) to adult and survival from nauplii to adult were the two response parameters, and the explanatory variables were temperature, food type, and salinity. Copepods undergo six nauplii and six copepodite stages where the sixth stage is the adult. Once per day, the number of living individuals and their life stage was observed. Individuals were classified as adults when females developed spike like extensions at the end of their prosome (one on each side of the urosome) and a distinct furca, males when they developed wavelike antennas and a distinct long furca (Katona, 1971).

### 2.4 Statistical analyses of life‐history traits

All analyses of data were done with R (R Core Team, 2019) and all figures by using the R package ggplot2 (Wickham, 2009). Development time and survival were analyzed in mixed models, functions lmer and glmer from the lme4 package (Bates, Mächler, Bolker, & Walker, 2015). Response variables were Gaussian for development time and binomial for survival. Fixed factorial effects for the models were the interaction of population and the experimental treatments, and family line was used as random effect. Treatment effects were analyzed as factors; thus, each factor combination represents a character state (Ghalambor, McKay, Carroll, & Reznick, 2007). Mixed model outputs were analyzed with type two ANOVAs using the car package (Fox & Weisberg, 2011). From the mixed models, a selection of contrasts between treatment combinations and associated *p*‐values are presented in the results. For contrasts of development time, the mixed model was fitted with function lme from the nlme package (Pinheiro, Bates, DebRoy, & Sarkar, 2017).

In the second experiment, the setup was not fully factorial, because the food type *Cryptomonas* sp. could not survive at 7 PSU, and hence, this treatment combination does not exist, and the interaction between population*temperature*salinity*food could not be tested. Therefore, the data were split in two analyses, one for population*temperature*salinity and one for population*temperature*food. The reason for not including both three‐way interactions in one model was that some factors would average over the uneven treatment. For example, the effect of salinity would compare the average of the two food types at 2 PSU with only one food type at 7 PSU. By dividing the data set into one for salinity and one for food type, two analyses of the main effects temperature and population, and the temperature*population interaction are presented in the results. However, both analyses led to the same conclusions, and both are presented in the results.

### 2.5 Broad sense heritability, genetic correlation, and interaction of genotype and environment

Heritability is a measure of the degree of resemblance between relatives; it aims to predict the phenotype of progeny from the phenotype of parents. In the context of heritability, an individual has two values, the phenotypic value, that is, the measured metric character, and the breeding value, that is, the average phenotype of its progeny expressed as deviations from the population mean (Falconer & Mackay, 1996). The phenotypic value is observable, but the breeding value is unobservable for the individual. The heritability provides a link from the selected phenotypes to the phenotype of the next generation. Hence, for selection and adaptive potential, the change in mean phenotype of a population has to be predicted from the correspondence between the parent phenotype and offspring. This is done by the breeder's equation: *R* = *h* ^2^ × *S*, where *R* is the response to selection, *h* ^2^ is the heritability and *S* the difference from the population mean to the mean of the selected individuals (Falconer & Mackay, 1996, eq. 11.2). The heritability is for a full sibling design estimated from the intraclass correlation: $t\;=\;\sigma_{B}^{2}/\left(\sigma_{B}^{2}+\sigma_{\varepsilon }^{2}\right)$ (Nakagawa & Schielzeth, 2010). Where $\sigma_{B}^{2}$ is the between‐group variation and $\sigma_{\varepsilon }^{2}$ is the Gaussian residual error variance, the heritability is then *t* ≥ 0.5 *h* ^2^ (Falconer & Mackay, 1996, table 10.2), where 0.5 is the average relatedness of full siblings.

The genetic correlation is similar to the heritability in the way that it estimates the link between phenotypic values and breeding values. However, here, the phenotypic value in one trait predicts the breeding value of the other trait. In the present study, the full siblings are crossed over temperature, and hence, it is possible to estimate the correlation of development time at different temperatures. Falconer (1952) and Yamada (1962) proposed that the same trait when measured in a different environment can be regarded as a different trait. This is because the physiology of the organism is expected to be different depending on environment and consequently also the genes required differ between the environments (Falconer & Mackay, 1996). The calculation of the correlation of the same trait at different temperatures is analogous to that of heritability as it is the correlation of between‐group variances at each temperature ${\text{COV}}_{\mathrm{XY}}/\sqrt{\sigma_{X}^{2}\times \sigma_{Y}^{2}}$ (Falconer & Mackay, 1996, eq. 19.2), where COV is the covariance of the families between two different temperatures (*X* and *Y*), and $\sigma^{2}$ is the between‐group variance of the families at a specific temperature (*X* or *Y*). The correlated response to selection (CR*_Y_*) is calculated as ${\text{CR}}_{Y}\;=\;ih_{X}h_{Y}G_{r}\sigma_{\mathrm{PY}}$ , where *i* the intensity of selection, $h_{X}$ and $h_{Y}$ are the heritability in the two environments, $G_{r}$ the genetic correlation, and $\sigma_{\mathrm{PY}}$ the standard deviation of the phenotypic value for character *Y* (Falconer & Mackay, 1996, eq. 19.6). The correlated, indirect, response to selection is weaker than direct selection; the two can be compared by $\frac{{\text{CR}}_{Y}}{R_{Y}}\;=\;G_{r}\frac{i_{X}h_{X}}{i_{Y}h_{Y}}$ (Falconer & Mackay, 1996, eq. 19.9).

The genotype by environment interaction and the genetic correlation are related in such a way that a specific configuration of reaction norms will lead to a specific correlation (Falconer, 1990). The genotype by environment interaction estimates the performance of each genotype, that is, family, from one environment to the next, and is as any interaction, a test of differences in slopes (Saltz et al., 2018). The variance of the family differences from the average reaction norm is the between‐group variance and creates a formal link between the interaction and the correlation (see results on genotype by environment interaction). For local adaptation, both estimates are fundamental as they describe how much of a phenotype is carried over from selection in one environment to its progeny in the next environment. In the present study, a short development time is assumed to be the best performance, and hence, a positive correlation between two environments would indicate that the best genotype in one environment also is best in the other environment. A negative or low correlation would indicate local adaptation and that selection has to be carried out in each environment separately to achieve the best performance.

Genetic correlations and broad sense heritability of development time were estimated by MCMC sampling using the function MCMCglmm (Hadfield, 2010). For genetic correlations and heritability, the unit of replication is at the family level; therefore, the data from both experiments were pooled to increase the precision of estimates. A very large number of replicates on family level are needed for any precise estimates of heritability and genetic correlations; this is typically not feasible in experimental studies and is instead more often available in animal breeding (Hoffmann, Merilä, & Kristensen, 2016). Nevertheless, an optimal design for heritability should reduce family size on behalf of a higher number of families. The optimal design is achieved when the sampling variance of the intraclass correlation is minimal, which it is when *n* = 1/*t* (Falconer & Mackay, 1996, chapter 10). However, *t* is not known before the experiment starts, and in the present study, the theoretical optimal family size was 1/*t* = 5.7, and the actual family size was on average 556/65 = 8.6 for the complete data set. Including larger families than the optimum is preferable as it is difficult to predict the percentage of individuals that will develop into adults beforehand, and hence, the resulting family size.

Five different estimates of genetic correlations between temperature treatments were calculated: between 12 and 17°C, 12 and 22.5°C, 17 and 22.5°C, GOR population at 17 and 22.5°C, and STHLM population at 17 and 22.5°C. The correlations of the same trait at different temperatures were estimated as the correlation of between‐group variances at each temperature. In MCMCglmm, this was set up as a bivariate model with the development time in the two temperatures as response variables. The models sampled the response variables respective variances in the posterior variance–covariance matrix (a 2 × 2 dimension matrix: $\sigma_{X}^{2}$ , ${\text{COV}}_{\mathrm{XY}}$ , ${\text{COV}}_{\mathrm{YX}}$ , $\sigma_{Y}^{2}$ ).

Twelve different estimates of heritability were calculated, two estimates for the complete data set and 10 estimates from subsets of the data set: 12, 17, 22.5°C, BB population, GOR population, GOR at 17°C, GOR at 22.5°C, STHLM population, STHLM at 17°C, and STHLM at 22.5°C. Heritability was not estimated for the BB population at the different temperatures and for the GOR and STHLM population at 12°C, because of a low number of replicates at family level. For heritability, the MCMCglmm model samples the posterior family variances and residual variances from which the intraclass correlation can be calculated. The heritability of a full sibling design can also be calculated within the framework of an ANOVA, see for example, Avery (2005) or Nakagawa and Schielzeth (2010) for calculations. The benefit of using MCMC within a random effects model is that the point estimates and uncertainties can be calculated directly from a large sample size of heritability estimates, that is, the posterior estimates. In addition, including fixed effect predictors in mixed models allows for estimation of adjusted heritability (e.g., Nakagawa & Schielzeth, 2010; Wilson et al., 2010). Although the intraclass correlation from the two different model frameworks should give similar results (Nakagawa & Schielzeth, 2010), a comparison of the heritability calculated from the complete data set is presented in the results.

For the correlation and heritability models, fixed effect covariates were included to control for the variance caused by the treatments and to avoid confounding effects on the between‐group variance and error variance (Nakagawa & Schielzeth, 2010). The models contained the following covariates: population, temperature, salinity, and food, when there was more than one treatment level per covariate.

For the MCMC models, inverse‐Wishart priors for the random effect were used; for heritability the variance was set to 2 and the belief parameter to 1 for the G‐structure (group), for the R‐structure (residual), respective values were 1 and 0.002. The belief parameter sets the values of the model parameters and describes the shape of the prior distribution. In the context of a mixed model, a group contains observations that are not independent, that is, the different full sibling families make up unique groups. For genetic correlations, the prior variances were set as the true variance for each trait (development time at a specific temperature) and the belief parameter to 3 (i.e., n dimensions of the G matrix + 1) (Hadfield, 2019; Wilson et al., 2010). The models ran for 2.6 million iterations with a burn‐in of 600,000 and sampled every 1,000 iteration, which generated an effective sample size of 2,000. From the 2,000 samples, the median and 95% quantiles (0.025, 0.975) are presented for heritability, and for genetic correlations, the mode and the 95% highest posterior density are presented.

The significance of the genotype by environment interactions was tested by model comparison in an analysis of deviance. One model with the temperature + family was compared with a model with the additional temperature*family term. The models were simple linear regressions with Gaussian error distribution; significance was assessed by F‐ratio tests. Furthermore, the variances of the fixed effect temperature and the random effect family across temperature (temperature|family) were quantified and compared by linear mixed models (lme). The analysis of deviance tests whether the reaction norm slopes are different for the families, whereas the mixed models quantify the variances of the overall effect of temperature and the variance of families across temperature (Bolker et al., 2009). Thereby, both the magnitude and the significance of the genotype by environment interaction were compared across temperature. The genotype by environment interactions was estimated for the same set of conditions as for the genetic correlations.

## 3 RESULTS

### 3.1 Environmental conditions

**Figure 2:**
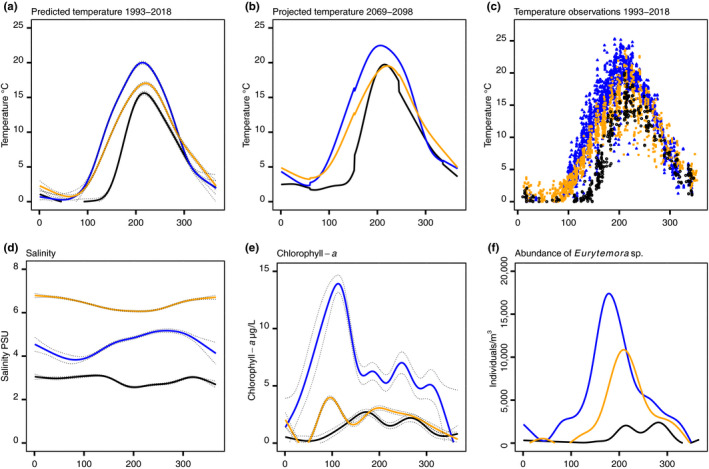
Predicted and projected environmental conditions for the sampling stations in the Baltic Sea for sea surface temperature (a), projected temperature (b), individual temperature observations (c), salinity (d), chlorophyll‐*a* (e), and abundances of all life stages (f), including nauplii, of *Eurytemora* sp. for the time period 1993–2018, except for (b) showing projected values for the years 2069–2098 (Meier, 2015). Panels (a) to (e) show observations from ≤10 m depth. The different sampling locations in the Baltic Sea are color coded: BB in black circles, GOR in blue triangles, and STHLM in orange squares. Lines are predicted fitted values from generalized additive models, and dotted lines are the respective 95% CI. The *x*‐axis in each panel shows the day of year

Long‐term surface temperature, projected temperature increase, chlorophyll‐*a*, and salinity differed among the three locations where the populations were sampled (Figure 2). The population size of *Eurytemora* sp. peaked during the summer months at all stations. For example, on August 7, average temperature and chlorophyll‐*a* were highest at the GOR site (19.9°C ± 0.21 CI and 5.4 µg/L ± 0.69 CI), intermediate at the STHLM site (17°C ± 0.21 CI and 2.9 µg/L ± 0.17 CI), and lowest at the BB site (15.6°C ± 0.25 CI and 1.5 µg/L ± 0.26 CI). Salinity differed between the stations the year round and was relatively stable compared with temperature and chlorophyll‐*a* (Figure 2) and was for example on August 7, the highest at the STHLM (6.1 PSU ± 0.03 CI), intermediate at the GOR (5 PSU ± 0.06 CI), and lowest at the BB (2.6 PSU ± 0.04 CI) site. The highest increase in temperature is predicted in the northern Baltic Sea (Meier, 2015), in year 2069–2098; this will result in similar maximum summer temperatures between BB and STHLM, 19.7 and 19.5°C, respectively, but temperature will remain the highest in GOR, 22.5°C (Figure 2).

### 3.2 Development time

**Figure 3:**
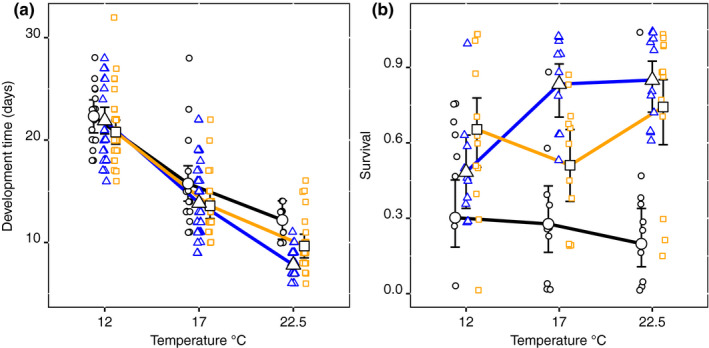
Development time from nauplii to adult (a) and survival from nauplii to adult (b) across temperature for the three Baltic Sea *Eurytemora affinis* populations. Estimates and 95% confidence intervals are from the model output. Points show the development time of individuals in (a) and average survival per family in (b). BB in black circles, GOR in blue triangles, and STHLM in orange squares

Differences in the populations’ development time depending on temperature were found in both experiments (Table 2). In the first experiment, where development time of all three populations across temperature was compared, the interaction of population and temperature was significant (*F*_4,256_ = 3.61, *p* = .007). Contrasts from the mixed model showed that all populations differed at 22.5°C (GOR vs. BB and STHLM: *t* = 3.97, *p* < .001; *t* = 2.28, *p* = .029; and BB vs. STHLM: *t* = −2.27, *p* = .030). At 22.5°C, the GOR population had the shortest development time with 7.8 days (6.7, 8.9; 95% CI), STHLM intermediate with 9.7 days (8.4, 10.8), and BB the longest with 12.2 days (10.3, 14.1) (Figure 3a). At 12 and 17°C, there were no significant differences between populations. Development time averaged over all populations was 21.7 days (20.9, 22.5) at 12°C and 14.2 days (13.5, 15.0) at 17°C.

**Table 2:** Analysis of variance output with type II sums‐of‐squares. Development time data was analyzed with *F*‐ratio tests and survival data with chi‐square tests. To simplify the presentation of the results, the models contained all the possible interactions, and omission or inclusion of nonsignificant terms did not affect the interpretation of the remaining effects

Experiment 1	Development time			Survival		
	*F*‐value	*df*	*p*(>F)	χ^2^	*df*	*p*(>χ^2^)
Population	5.05	2, 33	**.012**	38.22	2	**<.001**
Temperature	382.77	2, 252	**<.001**	5.05	2	.08
Pop.*Temp.	3.61	4, 256	**.007**	24.28	4	**<.001**
Experiment 2						
Interaction with salinity, *R. nottbecki* only						
Population	22.81	1, 25	**<.001**	0.19	1	.666
Salinity	74.02	1, 170	**<.001**	5.53	1	**.019**
Temperature	222.1	1,166	**<.001**	1.86	1	.173
Pop.*Sal.	12.75	1, 170	**<.001**	1.19	1	.276
Pop.*Temp.	0.05	1, 166	.815	0.13	1	.72
Sal.*Temp.	1.22	1, 166	.271	0.64	1	.426
Pop.*Sal.*Temp.	0.59	1,167	.445	1.06	1	.304
Interaction with food, 2 PSU only						
Food	274.82	1, 170	**<.001**	9.84	1	**.002**
Population	7.07	1,25	**.014**	1.88	1	.171
Temperature	186.68	1,169	**<.001**	0.22	1	.64
Food*Pop.	0.62	1, 170	.432	0.96	1	.328
Food*Temp.	0.1	1, 172	.75	5.79	1	**.016**
Pop.*Temp.	1.9	1, 170	.17	0.25	1	.615
Food*Pop.*Temp.	1.57	1, 172	.212	1.02	1	.314

**Figure 4:**
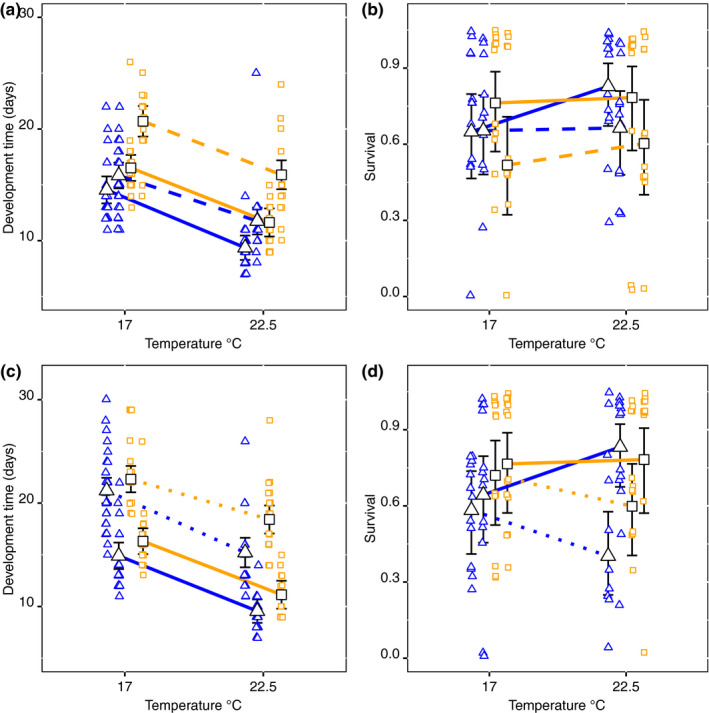
Development time from nauplii to adult (a, c) and survival from nauplii to adult (b, d) across temperature for the two *Eurytemora affinis* populations, GOR in blue triangles and STHLM in orange squares. The two upper panels show the interactive effect of population*temperature*salinity, and the dashed line shows 7 PSU and the solid line 2 PSU. The two lower panels show the interactive effect of population*temperature*food, and the dotted line shows *Cryptomonas* sp. and the solid line *R. nottbecki*. Estimates and 95% confidence intervals are from the model output. Note that the estimates for salinity 2 PSU and the food *R. nottbecki* are identical in (a, c) and (b, d), see methods

In the second experiment, the GOR and STHLM populations were crossed over temperature*salinity and temperature*food (Table 2, Figure 4a,c). The GOR population had in general shorter development time than the STHLM population, both when averaged over temperature and salinity (*F*_1,25_ = 22.81, *p* < .001) and over temperature and food (*F*_1,25_ = 7.07, *p* = .014). Development time was 12.6 days (11.6, 13.5; 95% CI) for the GOR population and 15.6 days (14.6, 16.7) for the STHLM population when averaged over temperature and salinity. Averaged over temperature and food development time was 14.5 days (13.3, 15.7) for the GOR population and 17.2 days (15.9, 18.5) for the STHLM population. The population*temperature interactions were not significant, neither when averaged over temperature and salinity (*F*_1,166_ = 0.05, *p* = .815) nor over temperature and food (*F*_1,172_ = 1.90, *p* = .170). Temperature had a significant effect on development time, both when averaged over population and salinity (*F*_1,166_ = 222.10, *p* < .001) and over population and food (*F*_1,169_ = 186.68, *p* < .001). Development time was at 17°C 15.8 days (16.8, 17.7) and at 22.5°C 11.0 days (12.0, 12.9) when averaged over population and salinity. Averaged over population and food, it was 17.7 days (18.8, 19.9) at 17°C and 11.9 days (13.0, 14.2) at 22.5°C.

For salinity, development time was significantly longer at 7 PSU than at 2 PSU (*F*_1,170_ = 74.02, *p* < .001), averaged over both populations. The STHLM population was more sensitive to a salinity change than the GOR population, and the increase from 2 to 7 PSU leads to comparably longer development time for the STHLM population (*F*_1,170_ = 12.75, *p* < .001) (Figure 4a). Development time was for the GOR population at 7 PSU 14.0 days (12.9, 15.1; 95% CI) and at 2 PSU 11.3 days (10.3, 12.4), and for the STHLM population, the same estimates were 17.8 (16.5, 19.1) days at 7 PSU and 14.1 days (12.9, 15.2) at 2 PSU.

For the food types, development time was significantly shorter on a diet of *R. nottbecki* than on a diet of *Cryptomonas* sp. averaged over populations, 12.8 days (11.8, 13.7; 95% CI) versus 19.7 days (18.7, 20.7) (*F*_1,170_ = 274.82, *p* < .001) (Figure 4c). However, there was no significant effect of population*food interaction (*F*_1,170_ = 0.62, *p* = .432). The estimated effect of food was a 6.9 days (5.9, 8.0) increase in development time from *Cryptomonas* sp. to *R. nottbecki* and similar to that of temperature, which was a 5.8 days (4.6, 7.0) difference from 17 to 22.5°C, the effect of salinity was considerably smaller and 3.0 days (2.1, 4.0) increase from 2 to 7 PSU.

### 3.3 Survival

In the first experiment (Table 2; Figure 3b), there was no significant main effect of temperature on survival, but a significant interaction of population and temperature ( $\chi_{4}^{2}$ = 24.28, *p* < .001). Furthermore, survival was in general lower for the BB population compared with the GOR and STHLM populations ( $\chi_{2}^{2}$ = 38.22, *p* < .001).

In the second experiment (Table 2; Figure 4b,d), the main effect of temperature was not significant when averaged over food ( $\chi_{1}^{2}$ = 1.86, *p* = .173), nor when averaged over salinity ( $\chi_{1}^{2}$ = 0.22, *p* = .640). However, the temperature*food interaction was significant ( $\chi_{1}^{2}$ = 5.79, *p* = .016), where survival at 17°C was on a diet of *Cryptomonas* sp. 65% (52, 77; 95% CI) and on a diet of *R. nottbecki* 71% (57, 82). In contrast, at 22.5°C, the estimates for the same food types were 49% (36, 62) and 83% (69, 91). That is, survival decreased with *Cryptomonas* sp. when temperature increased from 17 to 22.5°C. The contrasts from the mixed model showed that the GOR population had significantly lower survival at 22.5°C with *Cryptomonas* sp. as diet compared to *R. nottbecki* with respective 40% (24, 58) and 88% (73, 96) survival (*z* = 3.85, *p* < .001), while at 17°C, there was no difference between food types. For the STHLM population, the type of food had no effect, and hence, the temperature*food interaction was mainly driven by the GOR population. Furthermore, the main effect of salinity on survival was significant ( $\chi_{1}^{2}$ = 5.53, *p* = .019), and survival was higher at 2 PSU where it was 76% (66, 83) than at 7 PSU where it was 62% (51, 71).

### 3.4 Genotype by environment interaction, genetic correlations, and broad sense heritability

**Figure 5:**
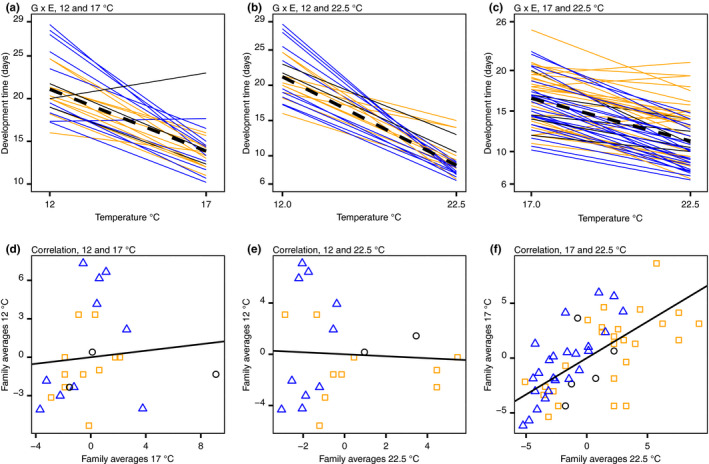
Development time from nauplii to adult for the different *Eurytemora affinis* populations: BB in black circles, GOR in blue triangles, and STHLM in orange squares for the different temperature combinations indicated in the panel titles. The upper panels (a), (b), and (c) show development time as reaction norms between temperatures, where the dashed lines show the average development time at each temperature. The lower panels (d), (e), and (f) show the same data as the panels above, but here as correlations between temperatures of the mean centered family averages with regression lines. The variances of the mean centered family averages are the between‐group variances that were used to estimate heritability and genetic correlations. The number of families and individuals in each panel is as in Figure 6b for the same correlation/reaction norm

**Figure 6:**
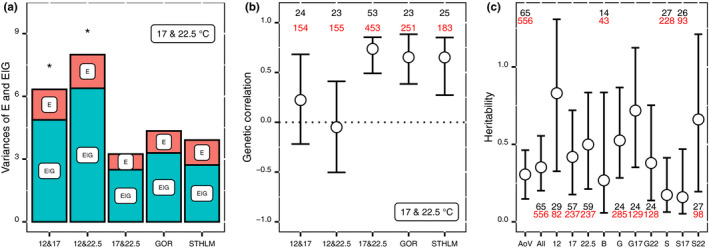
Panel (a) shows the environmental (E) and genotype by environment (E|G) variances expressed as standard deviations for the reaction norms between temperatures. Stars show significance of the genotype by environment interaction presented in Table 3, and the estimates for the GOR and STHLM populations are from 17 to 22.5°C. Panel (b) shows genetic correlations with highest posterior mode estimates and 95% credible intervals of development time from nauplii to adult for Baltic Sea *Eurytemora affinis*. The number of families and individuals is the same in (a) and (b). The three correlations to the left were calculated on the pool of the populations (BB, GOR, and STHLM), and the correlations for the GOR and STHLM populations were calculated between 17 and 22.5°C. In panel (c) from left to right: heritability for the complete data set at the three different temperatures from a one‐way ANOVA and a MCMCglmm, respectively, BB population, GOR population, GOR at 17°C, GOR at 22.5°C, STHLM population, STHLM at 17°C, and STHLM at 22.5°C. For the ANOVA estimate, errors are 95% confidence intervals, for the remaining estimates they are 95% credible intervals and the estimates are median values. For each estimate of genetic correlations (b) and heritability (c), the number of families (black) and individuals (red) for the respective estimate is given

Genotype by environment interaction was significant between 12 and 17°C, and 12 and 22.5°C but not significant between 17 and 22.5°C (Table 3, Figure 5). The results from the analysis of deviance were in agreement with the results from the linear mixed models. That is, when the genotype by environment interactions were significant, the variance of the interaction was also greater, and hence, there was more variation in phenotypic plasticity (Figure 6a). The variance in phenotypic plasticity was greater between the coldest temperature 12°C and the two higher temperatures (17 and 22.5°C), than between the two higher temperatures (Figure 6a).

**Table 3:** Analysis of deviance output with type II sums‐of‐squares for genotype by environment interactions. Models with and without the interaction term were compared between the temperatures stated in the “model” column. *p*‐values were calculated as *F*‐ratio tests on the difference in deviance and degrees of freedom between models

Model	Resid. *df*	Resid. deviance	*df*	Deviance	*F*	*p*(>*F*)
12 & 17°C						
Temp.+Family	129	1,295.2				
Temp.*Family	106	799.8	23	495.39	2.85	**<.001**
12 & 22.5°C						
Temp.+Family	131	1,139.4				
Temp.*Family	109	494.3	22	645.1	6.46	**<.001**
17 & 22.5°C						
Temp.+Family	408	4,422.8				
Temp.*Family	354	3,800.5	54	622.3	1.07	.346
17 & 22.5°C GOR						
Temp.+Family	232	2,119.0				
Temp.*Family	209	1,921.5	23	197.5	0.934	.553
17 & 22.5°C STHLM						
Temp.+Family	161	2,025.4				
Temp.*Family	136	1,713.5	25	311.9	0.990	.484

Genetic correlations between temperature treatments were not significantly different from zero between 12 and 17°C and between 12 and 22.5°C, while the correlation between 17 and 22.5°C was significantly positive (Figure 6b). The 95% credible intervals did not overlap for the correlations between 12 and 22.5°C and between 17 and 22.5°C, indicating that these two correlations are different. Correlations were significantly positive for the STHLM and GOR populations from 17 to 22.5°C (Figure 6b).

The estimated median values of heritability ranged from 0.16 to 0.83, and the 95% credible intervals for each estimate overlapped, indicating that there were no significant differences between the experimental conditions (Figure 6c). Heritability for the complete data set with covariates for population, temperature, food, and salinity had the most precise estimate 0.35 (0.20, 0.55; 95% CI). The heritability gives an estimate of the direct response to selection and the genetic correlations an indirect one. The ratio between direct and indirect selection was calculated between 17 and 22.5°C, by using the equation given in the methods and assuming the same intensity of selection (*i*) at both temperatures, *h* ^2^ = 0.42 at 17°C, *h* ^2^ = 0.5 at 22.5°C, and $G_{r}$ = 0.74. If selection occurs at 17°C, the correlated response is 0.62 at 22.5°C, and if selection instead occurs at 22.5°C, the correlated response is 0.88 at 17°C. Similarly, if selection is at 12°C, the indirect response at 22.5°C would be −0.03, and hence, a very weak antagonising effect, the sign, whether positive or negative, is however not significant between 12 and 22.5°C. These values are in proportion to direct selection, that is, if selection occurs separately at each temperature (see methods).

## 4 DISCUSSION

This study explores the selection and adaptation potential to changing environmental conditions of the copepod *E. affinis*, a key zooplankton species in coastal waters and in the Baltic Sea. We found *E. affinis* to be adapted to different temperature regimes and that the species can adapt to higher temperature than present via indirect selection at 17°C, which can result in an adaptation at 22.5°C. However, our results suggest that the adaptation to high temperature is unlikely to occur for populations located in “colder” temperatures, that is, 12°C. Global warming coupled with changes in food conditions and salinity may alter temperature tolerance, and the benefits of temperature adaptations may be compromised if additional changes in salinity and food conditions co‐occur.

Our results indicate that selection at a present temperature can facilitate adaptation to a more extreme future temperature. This because family lines that perform well at intermediate temperature will also perform well at higher temperature, indicated by the positive genetic correlation between 17 and 22.5°C, which confirm indirect selection, and hence, adaptive potential between the two temperatures (Figures 5 and 6b). The reaction norms of the genotype by environment interaction, E|G, between 17 and 22.5°C had in comparison lower variances than between the other temperatures (Figures 5 and 6a), indicative of overall low variance in phenotypic plasticity. Although low variance in phenotypic plasticity is typically seen as a limit of the evolutionary response (Dam, 2013; Ghalambor et al., 2007; Lee, 2002; Oostra, Saastamoinen, Zwaan, & Wheat, 2018; Sgrò, Terblanche, & Hoffmann, 2016), it is possible to see its potential benefits because all genotypes are more prone to respond similarly to both direct and indirect selection, and a short development time is likely beneficial at both 17 and 22.5°C. Hence, the interaction is not adaptation potential per se, as a significant genotype by environment interaction can result in antagonising selection as well. Therefore, the configuration of reaction norms, which determines the sign and strength of the genetic correlation, should preferably be considered together with the genotype by environment interaction to assess adaptive potential. In contrast, variance in the reaction norms between the cold (12°C) and the highest temperature (22.5°C) was greater and the genetic correlations indicated that indirect selection of development time at 12–22.5°C is unlikely (Figures 5 and 6ab). Hence, selection on a genotype with a shorter development time compared with the population mean at 12°C will likely have no effect on the development time at 22.5°C.

The populations had different development time at the highest temperature treatment with the GOR population having the shortest, STHLM intermediate, and BB the longest. Long and warm summers create better opportunities for adaptation to warm temperatures. The warm summer season is the time when abundances are the highest and consequently genotypes compete via their population rate of increase, and hence, they benefit by having as short generation times as possible. The development time in the present study was ordered as GOR<STHLM<BB, and temperature and chlorophyll‐*a* from the sites are ordered as GOR>STHLM>BB. For zooplankton, higher temperatures and more food lead to a shorter development time (Ban, 1994; Gillooly, 2000). Thus, the population from high temperature and food availability had a shorter intrinsic development time compared with the populations originating from lower temperatures and poorer food conditions. Hence, the covariance of the populations’ environmental values and the populations’ genotypic values is positive and, therefore, indicative of a cogradient variation (Conover, Duffy, & Hice, 2009; Falconer, 1990). In addition, the results suggest that life in a cold environment constrain evolution of increased performance in a warm environment, that is, warm adaptation (Angilletta, Huey, & Frazier, 2009; Frazier, Huey, & Berrigan, 2006). That is an increased performance in high temperature of high temperature populations, while all populations whether from low or high temperatures have similar performance in low temperatures. Hence, high temperatures drive the differentiation. This means that northern most populations of Baltic Sea *E. affinis* would adapt to high temperature to a lesser degree unless temperature increase to the 17°C threshold level. Whether or not southern populations will replace the northern most population by then is difficult to predict as adaptation can be fast once temperature increases.

We found no main effect of temperature on survival, neither under the first experiment when all three populations were included, nor during the second experiment with the GOR and STHLM populations. Given that the range of the *E. affinis* species complex span ca 30 latitudes, from the Gulf of Mexico in the south to the Bothnian Bay in the north (Lee, 2016), their temperature tolerance is expected to be wide (Deutsch et al., 2008). However, different clades and populations may be locally adapted and exhibit differences in survival in relation to temperature, such as the GOR population, where survival was lower at 12°C than at 17 and 22.5°C as shown in the present study. The 50% lethal temperature level for *E. affinis* is 29.6°C (Hammock et al., 2016); temperatures that high are well above our experimental temperature and projections for the Baltic Sea (Meier, 2015). The BB population had overall lower survival than the GOR and STHLM populations; consequently, the longer development time of the BB population could be a result of suboptimal culturing conditions for this population. However, development time is measured on families that survive whereas survival is measured on all families and the mortality in one family has no direct relation to the development time of another family. In addition, the development time of BB families at 12 and 17°C is similar to both the GOR and STHLM populations, suggesting that the culturing conditions are not more of an artifact for the BB population than for the other populations.

The *Cryptomonas* sp. diet was of lower quality in comparison with *R. nottbecki* as treatments with the former food source resulted in longer development time and lower survival. Food quality typically varies among phytoplankton species (Lang, Hodac, Friedl, & Feussner, 2011) and between freshwater and marine species (Galloway & Winder, 2015), where freshwater species, such as *Cryptomonas* sp., tend to have lower quality compared with marine species. At high temperature, the GOR population develops to maturity in a shorter time and to a larger size than the STHLM population (Karlsson & Winder, 2018). The combination of large size at maturity and short development time is unusual among ectotherms. Compared between populations, more often a trade‐off of these two traits exists, where fast development comes with small size (Allan, 1976; Gillooly, Charnov, West, Savage, & Brown, 2002; Kingsolver & Huey, 2008; Merilä et al., 2004; Roff, 1980). Thus, the GOR population lacks this trade‐off and has compared with the STHLM population better values in two key fitness traits. However, the GOR population had lower survival at 22.5°C on a diet of *Cryptomonas* sp. compared with *R. nottbecki*, while there was no difference in survival between food types at 17°C, suggesting a trade‐off in development time and survival depending on the food‐temperature interaction. In comparison, for the STHLM population, where the individuals are smaller and development time longer, there was no difference in survival related to temperature and food type. This agrees with observations showing that populations with higher intrinsic growth rates are comparably more sensitive to food deprivation than populations with lower growth rates (Gotthard et al., 1994; Stoks et al., 2006). The faster development and growth at high temperature makes the GOR population the stronger competitor; however, when food quality deteriorates and temperature remains high, mortality increases compared with the STHLM population. This shows a complex interaction between intrinsic population level trade‐offs and environmental stressors that would not been visible with temperature as the only treatment effect.

We found a significant effect of salinity on development time at low salinity leading to a shorter development time for both the GOR and STHLM populations. This is in contrast with observations showing that freshwater conditions prolong development time for both freshwater and estuarine *E. affinis* populations (Karlsson et al., 2018; Lee et al., 2003). However, decreased salinity affects metabolic rates and ingestion rates of *E. affinis*, and freshwater tolerance increases if the copepods are exposed to sufficient food availability, as in our experiment (Hammock et al., 2016; Lee et al., 2013). Increased feeding rates may thus both shorten development time and increase freshwater tolerance (Ban, 1994; Lee et al., 2013). It is therefore possible that the lower salinity evokes a stress response that leads to increased feeding, which in turn leads to shorter development time in the GOR and STHLM populations when salinity was reduced from 7 to 2 PSU (Figure 4a).

The heritability of *E. affinis* development time calculated from the complete data set was estimated to be 0.35. Heritability is typically low for life‐history traits that have high impact on fitness, such as development time (Berger, Postma, Blanckenhorn, & Walters, 2013; Bradshaw, Holzapfel, Kleckner, & Hard, 1997; Sniegula et al., 2016), and gives a direct measure of how much development time can change from one generation to the next. It is difficult to predict whether adaptation will take place within populations or if populations can reproduce and mix, forming metapopulations on which selection can act on. However, as *E. affinis* consists of a cryptic species complex with distinct populations that may be reproductively isolated even when they are co‐occurring (Favier & Winkler, 2014; Lee, 2000), it implies that it is uncertain if adaptations can happen by crossings of populations from warm and cold environments. Crossing of populations would result in greater genetic variance and could speed up adaptations. For the Baltic Sea, it is to a large extent unknown which populations can interbreed. There is evidence of invasive populations from the North American east coast that are found in the shallow bays of the eastern Baltic Sea such as the Gulf of Finland and Gulf of Riga (Sukhikh et al., 2013, 2019). Reproduction between populations of the shallow benthic and pelagic populations in the Baltic Sea is not yet tested, and it is hence unknown if it occurs (Sukhikh et al., 2019). However, proximate populations from the North American east coast have been found to be reproductively isolated (Lee, 2000); adaptation to temperature, salinity, and food conditions in *E. affinis* is likely limited within cryptic species that inhabit different environments.

## 5 SUMMARY

Our study shows that selection of development time at warmer temperatures of 17 and 22.5°C is positively correlated, and hence, *E. affinis* can adapt to higher temperatures if they currently inhabit waters of ≥17°C because of indirect selection that reinforce adaptation to high temperatures. In contrast, selection at cold and warm temperature was uncorrelated, and a population at 12°C is unlikely to adapt to 22.5°C. In agreement with the sign of the genetic correlations, we found that the population from the warmest site of origin had comparably shorter development time at high temperature than the populations from colder sites. This indicates that populations are locally adapted and have a cogradient variation of development time in response to temperature for this Baltic Sea copepod. Furthermore, we present a cost of short development time, where the fastest developing population had lower survival caused by a change in diet at high temperature, in comparison with a population with longer development time. This emphasizes the importance of including multiple environmental stressors and locally adapted populations to enhance our understanding of the effects of global change.

## CONFLICT OF INTEREST

The authors declare no conflicts of interest.

## AUTHOR CONTRIBUTIONS

MW and KK: designing and writing. KK: performing the experiments, maintaining cultures of organisms, data analysis, and writing (first draft).

## AUTHOR CONTRIBUTION

KK: Conceptualization (equal); Data curation (lead); Formal analysis (lead); Investigation (equal); Methodology (equal); Validation (lead); Visualization (lead); Writing‐original draft (lead); Writing‐review & editing (lead). MW: Conceptualization (equal); Formal analysis (supporting); Funding acquisition (lead); Investigation (equal); Methodology (equal); Project administration (lead); Resources (lead); Supervision (lead); Validation (supporting); Visualization (supporting); Writing‐original draft (supporting); Writing‐review & editing (supporting).

## Data Availability

Data and code to conduct the analyses and figures presented here are available from the Dryad repository https://doi.org/10.5061/dryad.rv15dv456
